# The Combination of Safety, Attractiveness, and Accessibility Lead to Bias in Inventory of Wetland Plants on the Qinghai‐Tibet Plateau

**DOI:** 10.1002/ece3.71521

**Published:** 2025-06-04

**Authors:** Yigang Li, Fan Liu, Meiying Gong, Xing Liu, Changchun Li

**Affiliations:** ^1^ Hubei Key Laboratory of Resource Utilization and Quality Control of Characteristic Crops, College of Life Science and Technology Hubei Engineering University Xiaogan Hubei China; ^2^ Key Laboratory of Biodiversity and eco‐Environmental Protection on the Qinghai‐Tibet Plateau of Ministry of Education, College of Life Sciences Wuhan University Wuhan Hubei China; ^3^ Wuhan Botanical Garden, Chinese Academy of Sciences Wuhan Hubei China

**Keywords:** accessibility, attractiveness, collecting bias, collecting‐priority, inventory completeness, Qinghai‐Tibet plateau, safety

## Abstract

Assessments of inventory incompleteness often focus on terrestrial taxonomic groups, with less attention given to freshwater ecosystem groups. In this study, we constructed a wetland plant database for the Qinghai‐Tibet Plateau (QTP) and evaluated the inventory incompleteness of wetland plants. By combining three types of variables: safety, attractiveness, and accessibility, we identified the key drivers of inventory incompleteness and established priority areas for further investigation. The results showed that the inventory incompleteness of wetland plants on the QTP is relatively low, and the areas with incomplete inventories are mainly concentrated in the Himalayas and Hengduan Mountains. Safety variables emerged as the most important factors influencing inventory incompleteness, with landslide risk, slope, species richness, and livestock density identified as key variables affecting this incompleteness. We identified the southeastern Hengduan Mountains as a priority area for future wetland plant surveys. Specifically, the priority areas for future wetland plant collection are mainly distributed in the Qomolangma Region, the middle reaches of the Yarlung Zangbo River, the Nyenchen Tanglha Mountains–Eastern Himalayas, the transition zone between the Northern Tibetan Plateau and the Hengduan Mountains, and the Qionglai Mountains–Daxue Mountains–Shaluli Mountains. This study provides valuable references for future wetland plant sampling and conservation efforts on the QTP.

## Graphical abstract

Inventory incompleteness of wetland plants on the Qinghai‐Tibet Plateau is relatively low. Safety variables are the most important factors influencing inventory incompleteness. Landslide index, slope, species richness and livestock density are the main variables influencing inventory incompleteness.

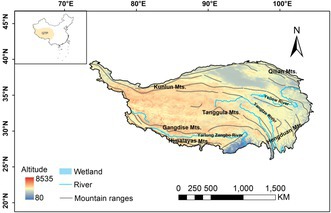

## 1 Introduction

A comprehensive and reliable dataset of species distributions forms the foundation for understanding large‐scale biodiversity patterns, predicting the impacts of global change, and identifying suitable conservation areas (Herrera‐R et al. 2023; Wang et al. 2020). However, comprehensive and accurate biodiversity data are still lacking in many regions and taxonomic groups (Aung et al. 2023; Herrera‐R et al. 2023; Troia and McManamay 2017). For instance, an assessment of plant collection completeness in the East African region revealed that 16% of the areas had no collections, while more than half exhibited incomplete collections (Wang et al. 2020).

The existence of biased and incomplete datasets significantly hinders a comprehensive understanding of biodiversity patterns. In regions where comprehensive species records are lacking, it becomes challenging to ascertain the true cause of a species' absence—whether it reflects a genuine absence or is merely a consequence of inadequate sampling. This limitation subsequently impacts our assessment of species diversity and the associated evolutionary relationships within the study area. For instance, a study examining plant collection patterns in Myanmar revealed a substantial gap in understanding the distribution of various plant taxa, particularly gymnosperms, pteridophytes, and bryophytes (Aung et al. 2023). Furthermore, inadequate species inventories also affect the analysis of correlations between species richness and turnover patterns across environmental gradients (Aung et al. 2023; Qian et al. 2018). Therefore, predicting species richness in a given region based on an existing species distribution dataset can offer a comprehensive assessment of geographic sampling bias in that region. Identifying the factors that influence this geographic bias can provide essential guidance for future species surveys and conservation research (Wang et al. 2020).

Research has shown that species sampling bias is influenced by a combination of factors (Wang et al. 2020). The following three hypotheses are currently receiving considerable attention. Firstly, survey accessibility, where it is generally accepted that more accessible sites, such as peripheral areas near roads, airports, and navigable rivers, will have higher sampling intensity (Herrera‐R et al. 2023; Yang et al. 2014). For example, road density and population density are the main predictors of sampling bias in fish sampling in Brazil (de Almeida et al. 2021). Population density and distance from cities are critical factors that significantly influence the integrity of freshwater fish surveys in Europe (Rodríguez‐Rey and Grenouillet 2022). Secondly, there is a phenomenon known as the “botanist effect,” whereby botanists tend to focus their attention on regions with higher levels of biodiversity or specific taxonomic groups (Herrera‐R et al. 2023; Yang et al. 2014). For example, protected areas are focal points for botanists due to their relatively undisturbed nature, pristine ecosystems, and high biodiversity (Herrera‐R et al. 2023; Yang et al. 2014). Thirdly, in terms of survey safety, although botanists tend to carry out extensive surveys, certain high‐risk environments may pose risks to personal safety and, consequently, hinder specimen collection efforts in these areas (Herrera‐R et al. 2023).

Species distribution models are powerful tools widely used in subjects such as biogeography and ecology. These models depend on available data regarding species distributions and are associated with environmental variables to predict the most suitable geographic ranges for species (Feeley and Silman 2011; Wang et al. 2020). With the availability of a large number of species occurrence datasets and environmental layers, the reliability of species range predictions has also improved (Mi et al. 2023). Therefore, predicting optimal distribution areas based on species distribution models and comparing these predictions with current species collection data can effectively assess the completeness of regional collections and thereby identify priority areas for future collection efforts (Troia and McManamay 2017; Wang et al. 2020).

**FIGURE 1:**
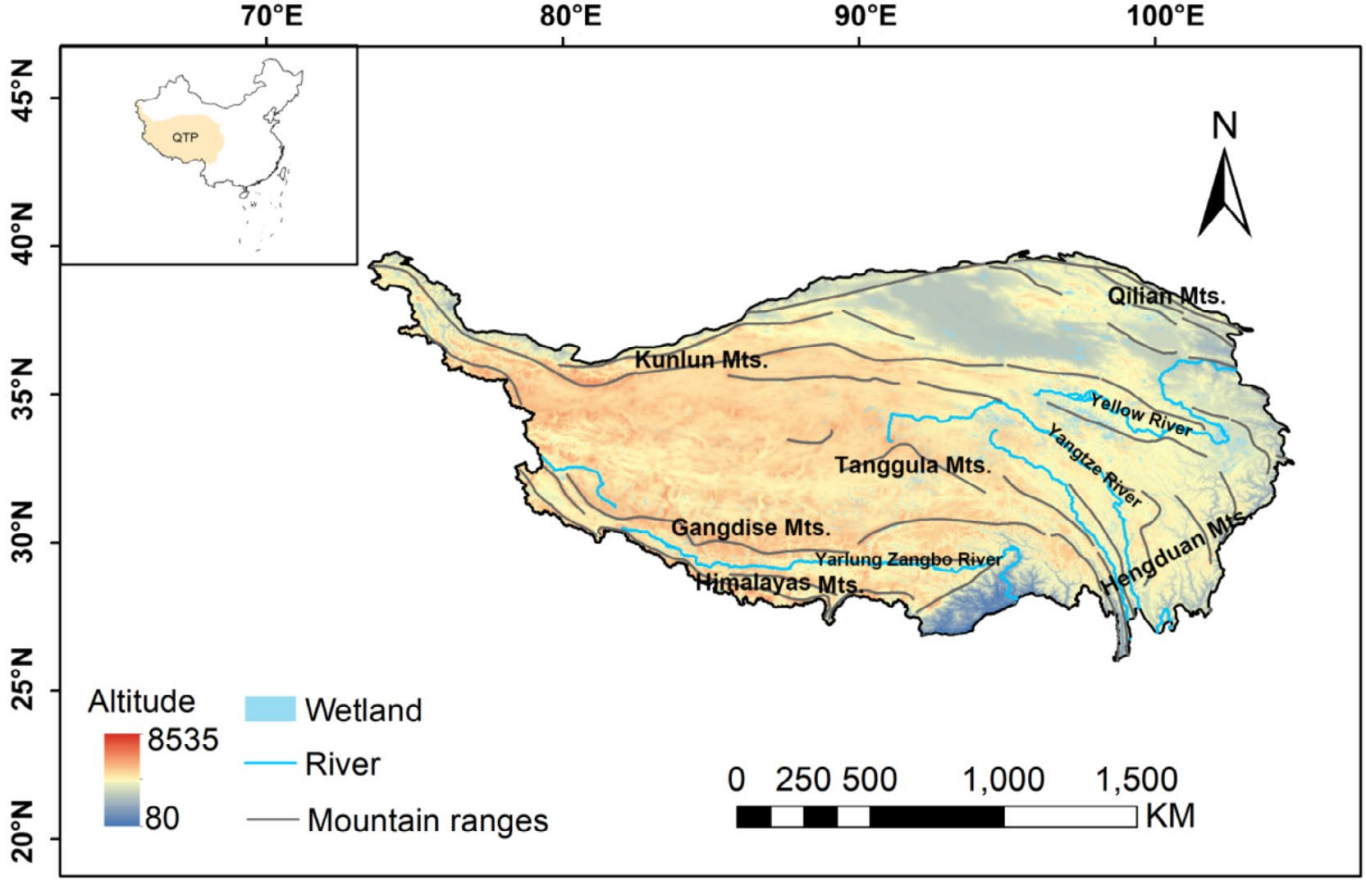
Geographic features and extent of the Qinghai‐Tibet Plateau.

The Qinghai‐Tibet Plateau (QTP) is home to the largest wetland groups in China (Zhao et al. 2015), with a relative abundance of wetland plants. Over the past 70 years, the country has established dozens of “national teams” and hundreds of “local teams,” and scientists have conducted in‐depth surveys and studies of the plant flora of the QTP, accumulating abundant plant specimen records, including wetland plants (Yan et al. 2013). The research results on the wetland plants of the QTP are concentrated in many important monographs and research papers. For example, China's Wetland Resources (State Forestry Administration 2015), Wetland Plants of Tibet (Liu, Xiao, et al. 2021), Atlas of Plant Identification in Maidica Wetland (La et al. 2022), Common Plants of Sanjiangyuan Wetland (Wei et al. 2018), List of Higher Plants in Datong River Basin of Qinghai (Zheng 2009), as well as research papers (Li et al. 2011, 2018; Wang 2003; Zhao 1994, 1997; Zhou et al. 2022). Therefore, the extensive data on wetland plant specimens collected by the QTP provides a solid basis for a comprehensive understanding and assessment of the completeness of wetland plant collections in the region (Figure 1).

Wetland ecosystems, serving as transitional zones between terrestrial and aquatic environments, harbor rich biodiversity and provide essential ecosystem services (Gao, Li, Brierley, et al. 2013, Gao, Li, Cheung, et al. 2013; Zhang et al. 2011). However, global climate change is accelerating the degradation of wetland ecosystems, particularly in the alpine wetlands of the QTP, which are highly vulnerable to the effects of climate change (Zhang et al. 2011). Wetland plants, which are important indicators of the health of wetland ecosystems, are also threatened by wetland degradation (Gao, Li, Brierley, et al. 2013, Gao, Li, Cheung, et al. 2013). Currently, numerous studies have focused on spatial biases in terrestrial biodiversity data (Farooq et al. 2021; Stropp et al. 2016; Wang et al. 2020; Yang et al. 2013), while research on freshwater biodiversity has been largely overlooked (de Almeida et al. 2021; Herrera‐R et al. 2023). In freshwater ecosystems, research on spatial gaps in inventory efforts is more prevalent for aquatic animal taxa (Belhaj et al. 2023; de Almeida et al. 2021; Herrera‐R et al. 2023; Sánchez‐Fernández et al. 2008), whereas studies on wetland plants remain relatively limited. Therefore, assessing inventory completeness for wetland plants on the QTP and identifying the drivers of sampling bias can provide a reference for future field surveys and conservation efforts on the plateau. Our study aims to (1) evaluate the completeness assessment of wetland plant specimen collections on the QTP and the geographic pattern of specimen collection bias; (2) seek the key factors driving the bias in wetland plant specimen collections based on accessibility, attractiveness, and safety; and (3) identify priority collection areas for the future based on the existing geographic pattern of collections.

## 2 Materials and Methods

### 2.1 Distribution Data for Wetland Plants

Using a large number of wetland plant monographs, online databases, literature sources, and field survey data (Appendix S1) (Li et al. 2024), we compiled a checklist of 1958 wetland plant species on the QTP, including100 aquatics and 1858 hygrophytes (Appendix S2). Species names were standardized mainly according to The Plant List version 1.1 (http://www.theplantlist.org), using the package “U.Taxonstand” (Zhang and Qian 2023). The distribution database of wetland plant species on the QTP was mainly obtained from the field surveys of 103 wetland sample sites and the Chinese Virtual Herbarium (CVH, http://www.cvh.ac.cn), the National Specimen Information Infrastructure (NSII, http://www.nsii.org.cn), the Global Biodiversity Information Network (GBIF, http://www.gbif.org), the Plant Photo Bank of China (PPBC, https://ppbc.iplant.cn/), and the literature. We conducted field surveys in July and August each year from 2018 to 2021. We established a total of 103 wetland sample sites with an altitude ranging from 1362 to 5157 m, a longitudinal range from 82.52° E to 100.35° E, and a latitudinal range from 27.46° N to 33.82° N. They were mainly located in the Hengduan Mountains region, the Yarlung Zangbo River basin, and the Qiangtang Plateau region, covering 35 counties. For each wetland sample site, the geographic coordinates, altitude, habitat information, and species information were accurately recorded. Three specimens of each species have been preserved for conservation and study. The distribution data were processed as follows: (1) Only field specimen records were retained, and records of species identified as introduced or cultivated in the QTP were deleted; (2) To ensure data adequacy and accuracy, in addition to specimen records with explicit latitude and longitude information that were directly included in the database, specimen records with geographic distribution information at the township, village, or more detailed level were also converted to latitude and longitude and included in the database using geocoding techniques; (3) Exotic, invasive, or artificial plants were excluded, and varieties and subspecies were merged into their respective taxonomic units at the species level (Zhou, Qian, Xiao, et al. 2023). As a result of the above standardization process, a total of 38,810 valid geographic distribution records were obtained.

### 2.2 Environmental Data for Species Distribution Modeling

In this study, the environmental data used to model species distributions fell into three main categories: climate data, topographic data, and land use data. The climate data included 19 bioclimatic variables obtained from the WorldClim 2.1 database (https://www.worldclim.org), as well as potential evapotranspiration (PET) and actual evapotranspiration (AET), with a resolution of 30 s. PET data were obtained from version 3 of the Global Aridity Index and Potential Evapotranspiration database, downloaded from the Plant Science Data Center (https://www.plantplus.cn/), while AET data were obtained from the CGIAR‐CSI Global Database (www.cgiarcsi.org). To avoid multicollinearity, Pearson correlation analysis was performed, and only variables with a correlation coefficient (*r*) below 0.8 were retained. The topographic data were divided into altitude and slope. Altitude was extracted from the SRTM 250 m DEM data downloaded from the Resource and Environmental Science Data Platform (https://www.resdc.cn/). Slope was extracted from the slope map of the Tibet Plateau downloaded from the National Tibetan Plateau/Third Pole Environment Data Center (https://data.tpdc.ac.cn/). China's Multi‐Period Land Use Land Cover Remote Sensing Monitoring Data Set (CNLUCC) was obtained from the Resource and Environmental Science Data Platform (https://www.resdc.cn/) (Xu et al. 2018). Finally, 10 climate variables and PET, altitude, slope, and land use were selected as the environmental predictors.

### 2.3 Construction of Species Distribution Modeling

Biomod2 is a widely used ensemble model for predicting species distributions. It effectively fits and compares various models, overcoming the limitations of individual models that exhibit low stability and significant bias (Gong et al. 2022; Hao et al. 2019; Record et al. 2013; Thuiller et al. 2009). Biomod2 has been employed to predict the most suitable geographic areas for species distribution across different geographic regions and taxonomic groups (Assefa et al. 2022; Li et al. 2022; Zhang et al. 2017; Zhao et al. 2021). In this study, we used the biomod2 package to create an integrated model for determining the optimal distribution of wetland plants, which combines 10 modeling algorithms (Assefa et al. 2022). To better simulate the actual distribution and reduce spatial bias, we randomly selected 500 pseudo‐absent points and repeated the modeling process three times. We used 75% of the random samples from the dataset as training data and reserved 25% for model evaluation (Zhao et al. 2021). The true skill statistic (TSS) was employed to assess predictive performance, retaining only models with a TSS ≥ 0.7 (Zhao et al. 2021). Individual models were weighted based on model performance (TSS) for each species to create an ensemble model (Mi et al. 2023; Zhao et al. 2021). We classified the habitat suitability zones of wetland plants into four levels: 0 to 0.05 as unsuitable zone; 0.05 to 0.33 as low suitability zone; 0.33 to 0.66 as medium suitability zone; and 0.66 to 1 as the most suitable zone (Zhang et al. 2017). In this study, we selected spatial cells with a probability of presence ≥ 0.66 as the suitable distribution area for wetland plants and transformed the potential distribution area into a presence/absence matrix. The suitable area is designated as the presence area (1), while the unsuitable area is designated as the absence area (0). The spatial pattern of the potential distribution of wetland plants was further analyzed based on the matrix (0, 1) (Zhao et al. 2021). To maintain the reliability of our distribution model, we only modeled species for which there were at least five wetland specimen records. For the remaining species, we documented their locations based on recorded specimen locations (Wang et al. 2020). To avoid overfitting the simulation due to clustering of wetland plant specimen distribution points, we used ENMTools V1.3 software to sparse the wetland plant specimen data. Finally, a total of 25,737 geographic distribution records were obtained for species distribution models.

### 2.4 Assessment of Inventory Incompleteness

We conducted a statistical analysis of species data based on grid units. For each grid unit, we counted the number of recorded plant species (*S*_original_) and calculated the potential number of wetland plant species predicted by the biomod2 distribution model (*S*_potential_). Species richness (*S*_r_) within a grid unit is defined as the sum of recorded species (*S*_original_) and predicted species (*S*_potential_), with each species counted only once. The formula for sample collection completeness is *V*_c_ = *S*_original_/*S*_r_ (Colwell and Coddington 1994). Therefore, the formula for inventory incompleteness is *V*_inc_ = 1–*V*_c_, where a larger value indicates a higher degree of incomplete collection.

### 2.5 Explanatory Variables for Inventory Incompleteness

In this study we analyzed three categories of variables that could potentially influence the collection of wetland plant specimens: accessibility, attractiveness, and safety. Accessibility consisted of road density (RDE), human population density (HD), road distance (RDI), and livestock density (LD). Attractiveness consisted of nature reserve area (NR), wetland area (WA), and predicted species richness (SR). Safety consisted of landslide risk (LR), slope (Slope), and inundation extent (IE). We downloaded road data for China from Open Street Map (https://www.openstreetmap.org/) and extracted road density (RDE) and road distance (RDI) using Arcgis 10.2. Road density (RDE) is defined as the ratio of the total length of roads in the grid to the area of the grid. As wetland data are represented as polygon vectors and road data as line vectors, the road distance (RDI) is defined as the shortest distance from the road line to any point on the wetland polygon. During field surveys, we found that some wetlands on the QTP are located within livestock grazing areas that are extensively fenced with wire barriers, making access impossible. Therefore, in this study we used livestock density (LD) as a proxy for accessibility. Livestock density data were obtained from the 2020 high‐resolution seasonal distribution dataset of livestock on the Qinghai‐Tibet Plateau. Population data were downloaded from the Resource and Environmental Science Data Platform (https://www.resdc.cn) (Xu 2017). In this study, we selected Landsat Tier 1 satellite images to extract the extent of surface water bodies in the QTP. A total of 123,461 Landsat 5, Landsat 7, and Landsat 8 images taken between 1991 and 2020 were analysed to calculate the frequency of water occurrence at the pixel level through the following three steps. First, we determined the number of valid pixel‐level observations of Landsat 5, Landsat 7, and Landsat 8 over the 30 years on the QTP. Valid observations are those that are not affected by clouds, cloud shadows, terrain shadows or snow cover. The quality assessment (QA) band generated by the Fmask algorithm was used to identify these factors. If the confidence level was high (> 66%), the observations were excluded (Hou et al. 2022). We then summed the valid observations for each pixel over the 30 years to create a layer of valid observations for the QTP. We then used the QA band to identify water bodies based on the valid observations and summed the water observations for each pixel over the 30 years to create a layer of water observations for the QTP. Finally, we calculated the percentage of water observations divided by the valid observations at the pixel level to determine the frequency of water occurrence (Pekel et al. 2016). In this study, water body frequency was categorised into two thresholds: 25% and 75%. A pixel with a water body frequency exceeding 75% is classified as a permanent water body, while a frequency between 25% and 75% is designated as a seasonal water body (Hao et al. 2023). Based on the water body frequency values of each pixel over 30 years, the average seasonal water body area and permanent water body area of the QTP were calculated. The inundation extent (IE) is defined as the percentage of the seasonal water body area relative to the total water body area (Herrera‐R et al. 2023). The data on nature reserves were obtained from the distribution pattern of terrestrial vascular plants and their conservation in the Qinghai‐Tibet Plateau (Liu et al. 2023). The landslide risk data was provided by Bintao Liu from the Institute of Mountain Hazards and Environment, Chinese Academy of Sciences. We used Pearson analysis to assess the correlations between all explanatory variables (Appendix S3), and employed ArcGIS 10.2 to plot the spatial pattern maps of the explanatory variables (Figure S1). In this study, both dependent and independent variables were transformed using log_10_(*n* + 1) transformations to obtain the best model fit and residuals that approximate a normal distribution. This approach was employed to mitigate extreme bias from values that deviate significantly from the mean (Ballesteros Mejia et al. 2013; Wang et al. 2020).

### 2.6 Identification of Priority Collection Areas for Wetland Plants

In this study, we identified priority survey areas for wetland plants by assessing measures of sampling incompleteness and species richness. We employed the methodology for delineating priority conservation areas and utilized weighted species richness of wetland plants to inform this analysis (Albuquerque and Beier 2015; Wang et al. 2020). Weighted species richness (WR) is calculated using the formula: $\mathrm{WR}=\sum_{1}^{n}\left(1/c_{i}\right)$, where *c*_*i*_ is the number of sites occupied by species *i*. The weighted species richness for a given site is summed over the *n* species occurring in the site (Williams et al. 1996; Zhou, Qian, Xiao, et al. 2023). The formula for calculating priority areas is *P* = WR × *V*_inc_ (Wang et al. 2020).

### 2.7 Data Analysis

In this study, we used a simple linear regression model to analyze the relationship between inventory incompleteness and the explanatory variables. All explanatory variables and inventory incompleteness were log_10_ (*n* + 1)‐transformed. The “calc. Relimp” function in the R package “relaimpo” was used to calculate the relative importance of the explanatory variables (Grömping 2006). Since spatial autocorrelation can influence the explanatory power of regression models, we utilized spatial simultaneous autoregressive error (SAR) models to further assess the relative importance of explanatory variables related to inventory incompleteness. To mitigate the effects of multicollinearity on the model, we excluded certain environmental factors with strong collinearity. Initially, ordinary least squares (OLS) regression was employed to identify the most suitable predictors. To ensure comparability of the model coefficients, all variables were standardized prior to model execution. From the various models constructed using possible combinations of environmental variables, predictors were selected utilizing the step() function in conjunction with Akaike's information criterion (AIC) (Burnham and Anderson 2002). SAR models were constructed using the predictor variables identified from the optimal model developed in Spatial Analysis in Macroecology (SAM) (Rangel et al. 2010). The relative importance of each environmental variable was determined using standardized regression coefficients in the SAR models. Finally, variance partitioning analysis (VPA) was applied to assess the relative importance of three categories of variables—accessibility, attractiveness, and safety—on the effect of inventory incompleteness. The R package “vegan” was used for VPA (Dixon 2003) and all data analyses, except for the SAR models, were carried out in R 4.3.1 (R Core Team 2017).

## 3 Results

### 3.1 Geographic Patterns of Inventory Incompleteness

**FIGURE 2:**
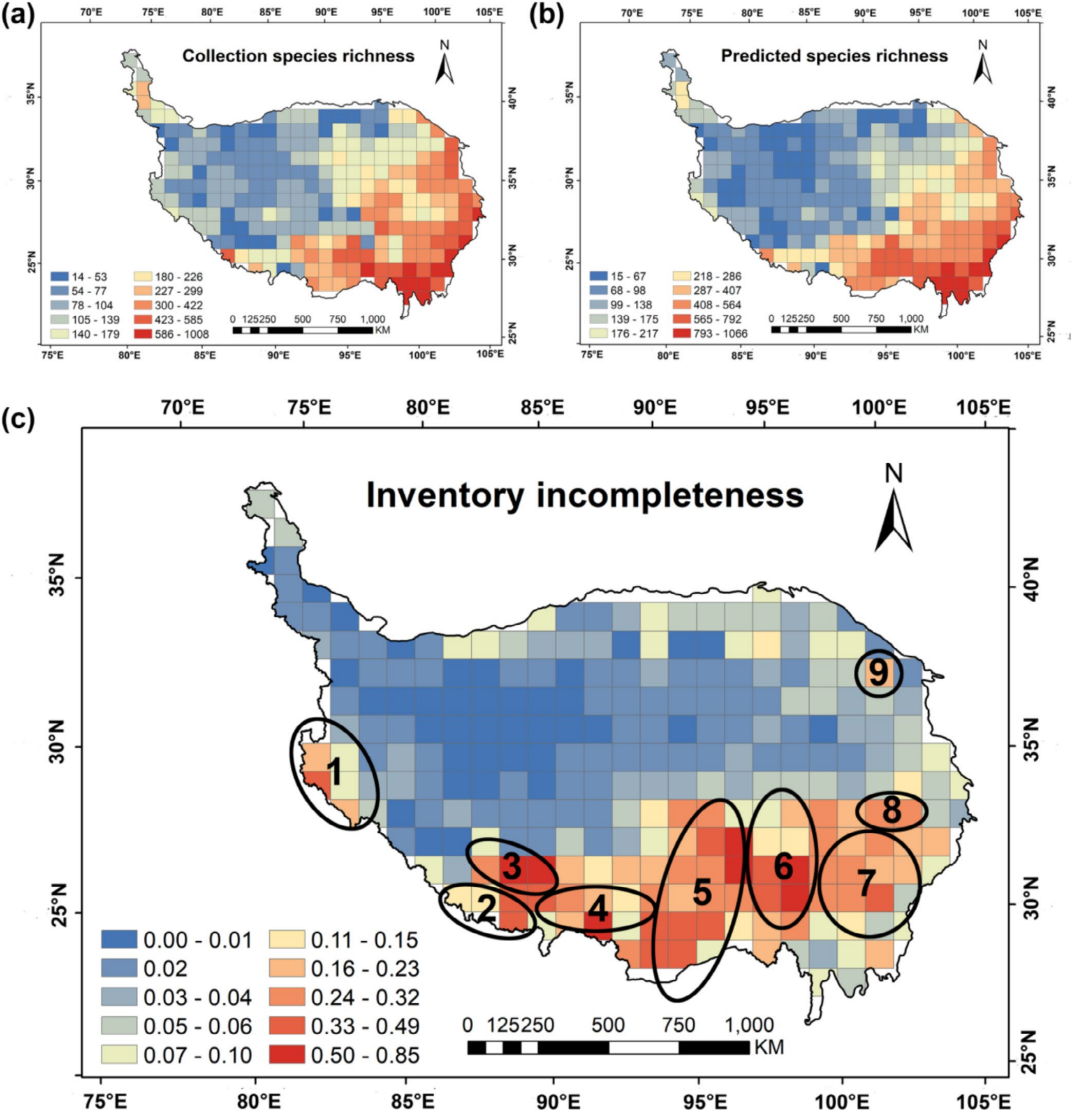
Geographic patterns of collection species richness (a), predicted species richness (b), and inventory incompleteness (c) for wetland plants on the Qinghai‐Tibet Plateau, classified using the Natural breaks (Jenks) method based on a 100 × 100 km grid. 1—Western Himalayas, 2—Qomolangma Region, 3—Gangdise Mountains, 4—Middle reaches of the Yarlung Zangbo River, 5—Tanggula Mountains (southeast)–Nyenchen Tanglha Mountains–Eastern Himalayas, 6—Transition zone between the Northern Tibetan Plateau and the Hengduan Mountains, 7—Qionglai Mountains–Daxue Mountains–Shaluli Mountains, 8—Bayankala Mountains (southeast), 9—Southeast branches of the Qilian Mountains.

**FIGURE 3:**
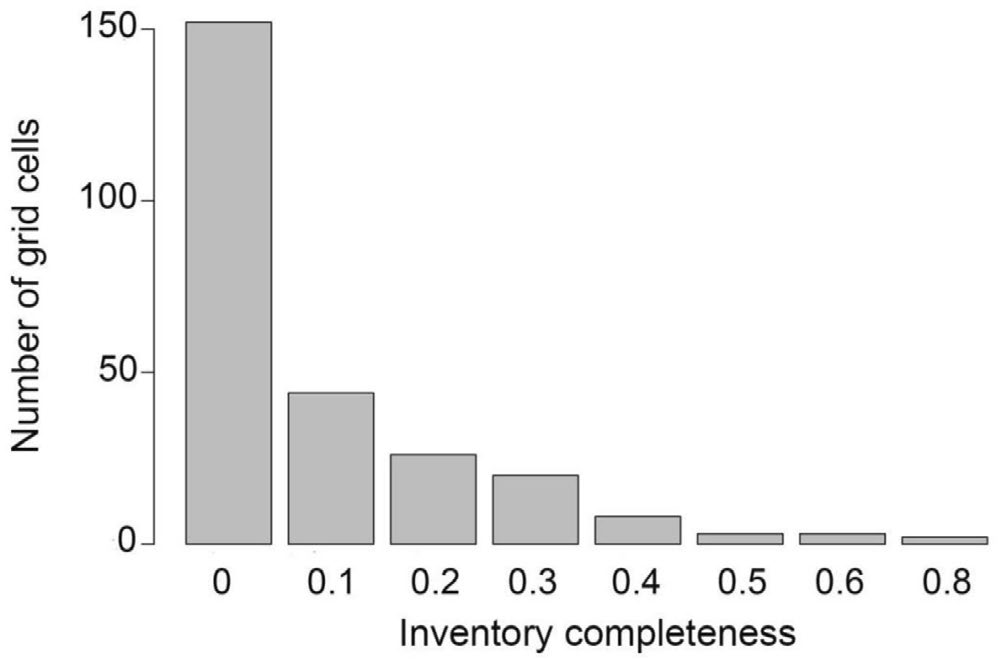
Statistics on the number of grids with inventory incompleteness.

The predicted species richness of wetland plants on the QTP ranged from 15 to 1064 per grid (Figure 2). Notably, the regions with high species richness were mainly concentrated in the Himalayas, the Hengduan Mountains, the Sanjiangyuan, and the Qilian Mountains (Figure 2), which were basically consistent with the geographic pattern of the actual species richness collected. The inventory incompleteness of wetland plants varied between 0 and 0.85, and the average value was 0.09. Among them, there were about 152 grids with inventory incompleteness of 0. The total number of grids with inventory incompleteness < 0.1 was 196, accounting for 75.97% of the total number of grids, while the number of grids with inventory incompleteness > 0.8 was found in only 2 grids, accounting for 0.78% of the total number of grids (Figure 3). This indicated that the integrity of wetland plant specimen collection on the QTP is very high. The results showed that the regions with higher inventory incompleteness are mainly located in the southern and southeastern parts of the plateau, such as the Himalayas, the Gangdise Mountains, the Yarlung Zangbo River, and the Hengduan Mountains region, while the other regions have lower levels of incompleteness (Figure 2).

### 3.2 Drivers of Inventory Incompleteness

**FIGURE 4:**
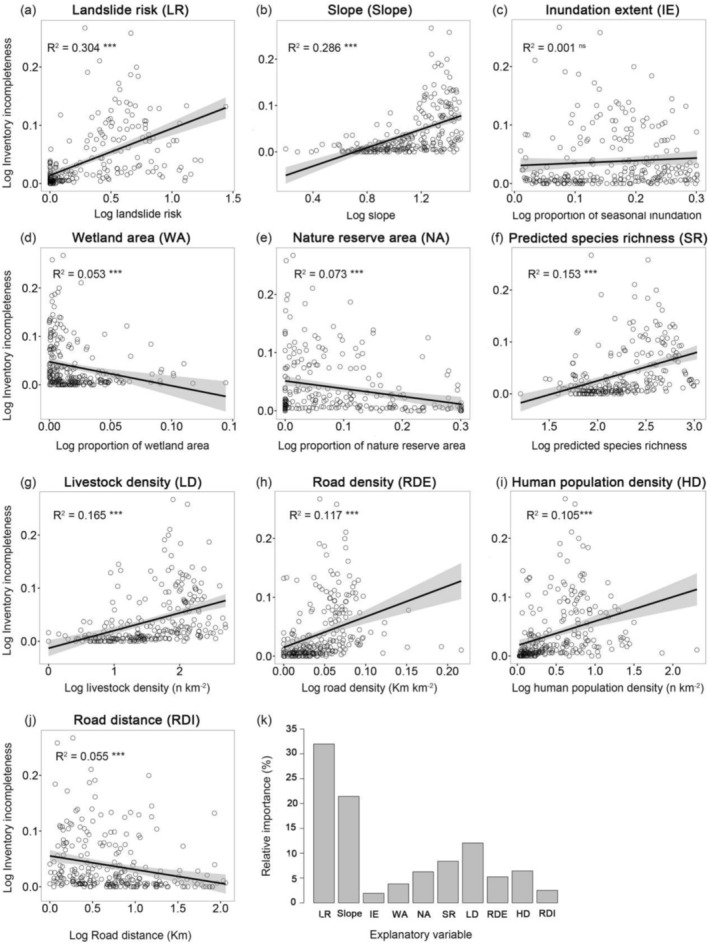
Single predictor relationships between explanatory variables and the incomplete collection of wetland plants on the Qinghai‐Tibet Plateau (a–j). The black lines represent the fit of the ordinary least squares (OLS) model, and the grey edges represent the 95% confidence intervals. The relative importance of each variable in relation to incomplete collection is explained (*k*). All explanatory variables and inventory incompleteness were log10 (*n* + 1)‐transformed. Significance: *** < 0.001; ** < 0.01; * < 0.05; ns not significant.

Analysing the results of the linear regression, we found that inventory incompleteness was significantly positively correlated with landslide risk, slope, and livestock density (*p* < 0.001; Figure 4a,b,g), and significantly negatively correlated with wetland area, nature reserve area, and road distance (*p* < 0.001; Figure 4d,e,j). However, there was no significant correlation with inundation extent (*R* ^2^ = 0.001; Figure 4c). Surprisingly, there was a significant positive correlation between inventory incompleteness and predicted species richness and human population density (Figure 4f,i). Among the explanatory variables ranked by their importance in relation to inventory incompleteness, landslide risk, slope, livestock density, and predicted species richness showed relatively high importance (Figure 4k).

**FIGURE 5:**
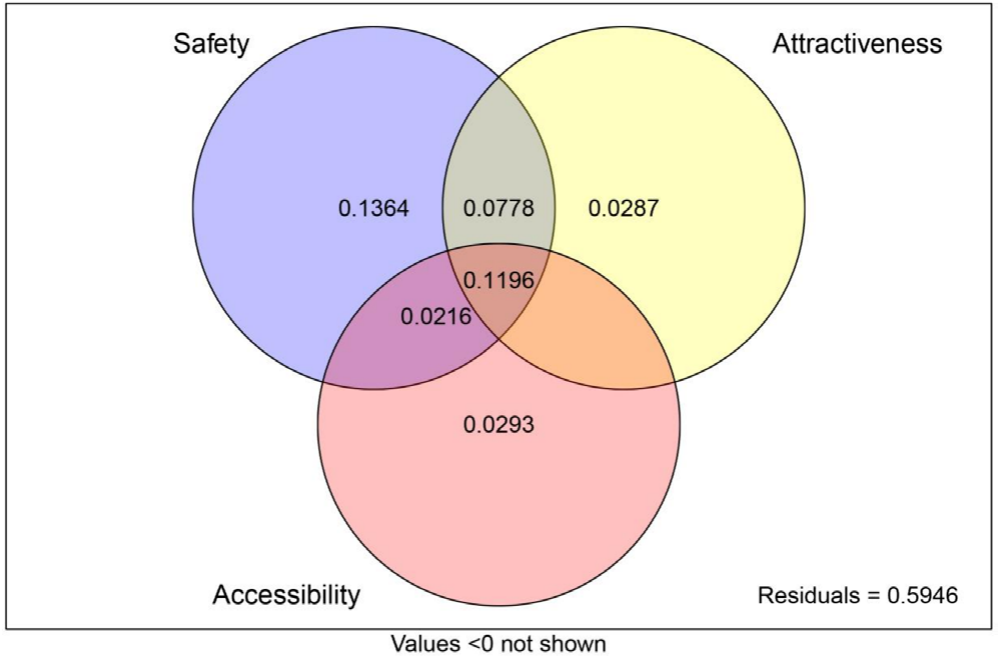
Variance partitioning (proportions) of inventory incompleteness into the independent effects of safety, attractiveness, and accessibility, as well as their overlap.

The OLS and SAR models explained 40.7% and 47.3% of the variance in inventory incompleteness, respectively (Table 1). The results showed that the relative importance of the explanatory variables obtained from the two models was relatively consistent, with landslide risk (LR), slope (Slope), predicted species richness (SR), human population density (HD), and livestock density (LD) being the most important explanatory variables. The results of the variance partitioning analysis indicated that the three types of variables together explain 40.54% of the variance in inventory incompleteness (Figure 5), with safety variables accounting for the highest explanatory rate at 35.54%. The attractiveness and accessibility variables can explain 22.61% and 17.05% of the variation in inventory incompleteness, respectively.

**TABLE 1:** Results of multiple linear regression (OLS) and spatial simultaneous autoregressive error (SAR) models analyses of inventory incompleteness for wetland plants.

	Inventory incompleteness			
	coef_ols_	*p*	coef_SAR_	*p*
Landslide risk (LR)	0.63	< 0.001	0.433	< 0.001
Slope (Slope)	0.325	< 0.001	0.343	< 0.001
Nature reserve area (NA)	−0.103	< 0.05	−0.08	> 0.05
Predicted species richness (SR)	−0.243	< 0.01	−0.288	< 0.01
Livestock density (LD)	0.189	< 0.05	0.189	< 0.05
Human population density (HD)	−0.343	< 0.001	−0.232	< 0.05
AIC	607.06		585.48	
*R* ^2^	0.407		0.473	

*Note:* The best OLS regression (with the lowest AIC) was selected from all possible combination models of the 10 explanatory variables. The explanatory variables used in the SAR model were consistent with the OLS model.

### 3.3 Identifying Priority Collection Areas

**FIGURE 6:**
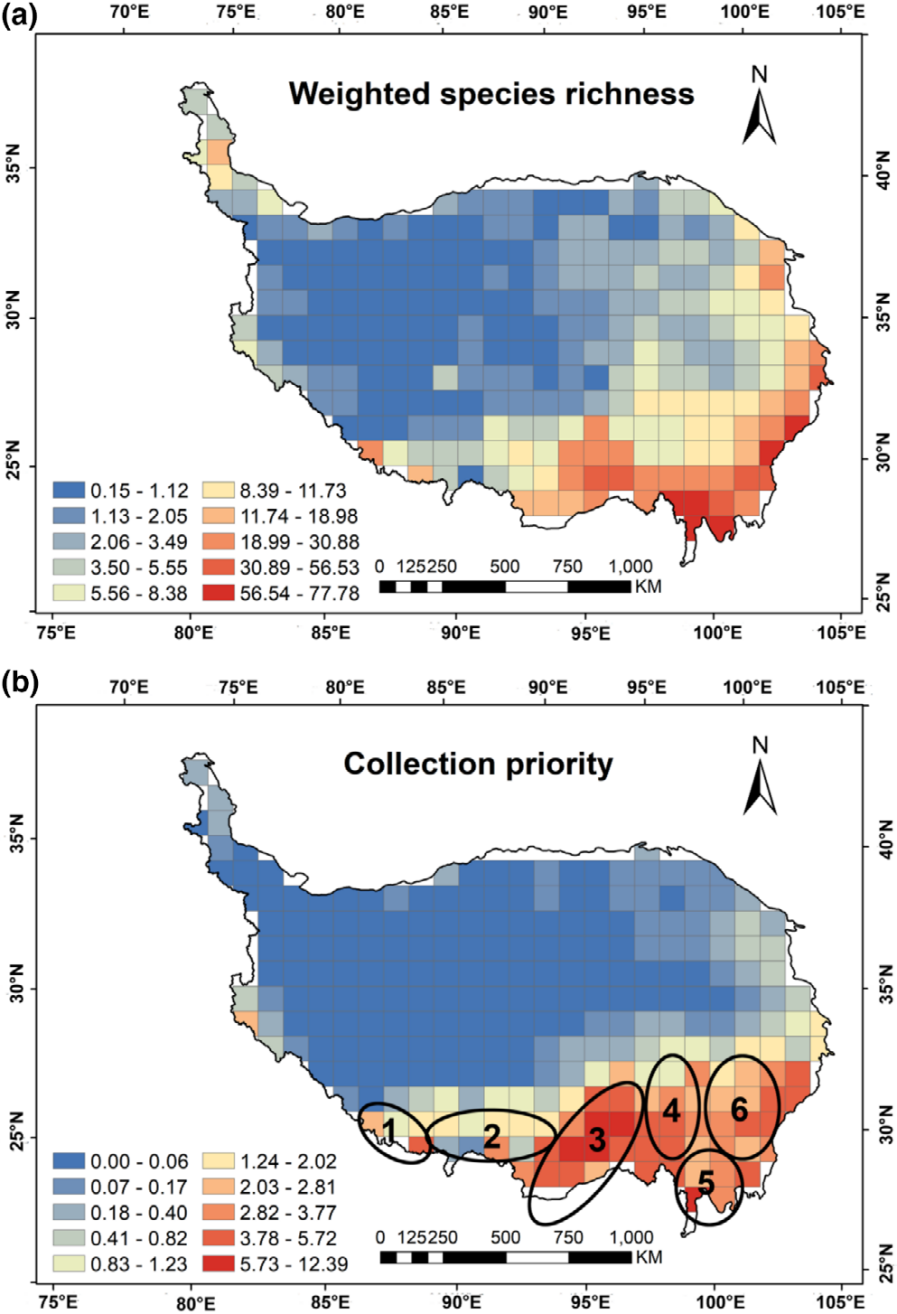
Geographic patterns of weighted species richness (a) and (b) collection priority, classified using the Natural breaks (Jenks) method based on a 100 × 100 km grid. 1—Qomolangma Region, 2—Middle reaches of the Yarlung Zangbo River, 3—Nyenchen Tanglha Mountains–Eastern Himalayas, 4—Transition zone between the Northern Tibetan Plateau and the Hengduan Mountains, 5—Qionglai Mountains–Daxue Mountains–Shaluli Mountains.

**FIGURE 7:**
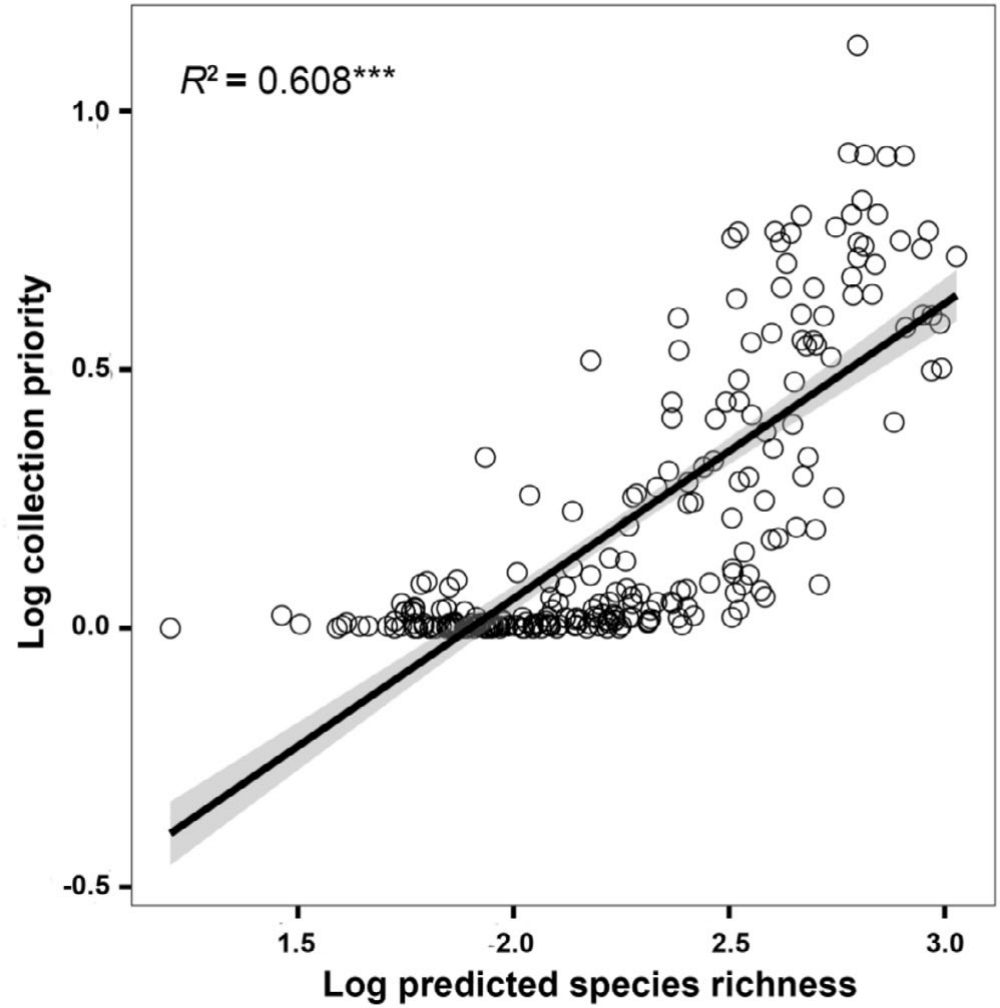
The linear relationship between predicted species richness of wetland plant and collection priority in the Qinghai‐Tibet Plateau. Significance: *** < 0.001; ** < 0.01; * < 0.05.

**FIGURE 8:**
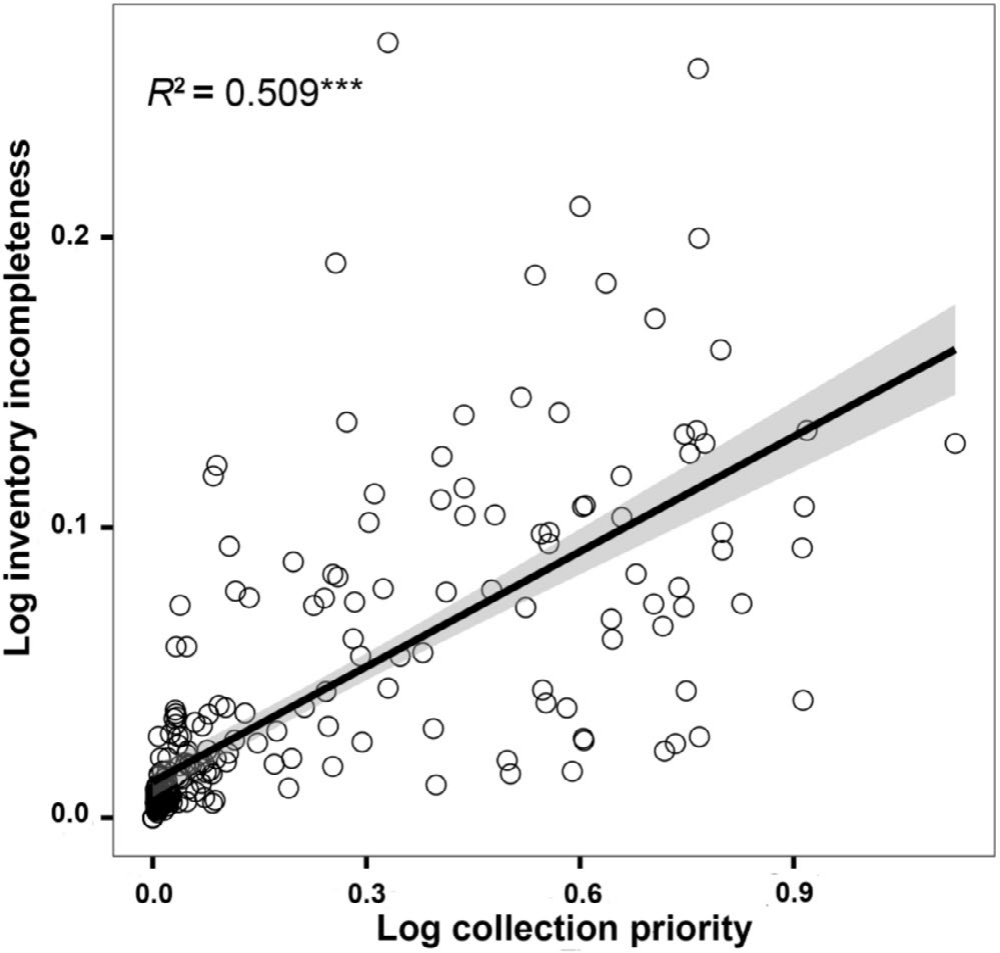
The linear relationship between collection priority of wetland plant and inventory incompleteness in the Qinghai‐Tibet Plateau. Significance: *** < 0.001; ** < 0.01; * < 0.05.

The weighted species richness of wetland plants ranged from 0.15 to 77.78 on the grid (Figure 6a). This measure was consistent with the overall trend of species richness; however, it specifically highlighted regions where plants exhibited narrower distribution ranges. The collection priority of wetland plants on the QTP ranged from 0 to 12.39, with an average of 0.97. The results showed that the priority areas for wetland plant collection were relatively consistent with the geographic pattern of collection incompleteness, mainly concentrated in the Himalayas in the south and the Hengduan Mountains in the southeast (Figure 6b). Specifically, the priority areas for future wetland plant collection are mainly distributed in the Qomolangma Region, the middle reaches of the Yarlung Zangbo River, the Nyenchen Tanglha Mountains—Eastern Himalayas, the transition zone between the Northern Tibetan Plateau and the Hengduan Mountains, and the Qionglai Mountains—Daxue Mountains—Shaluli Mountains (Figure 6b). A significant positive correlation was found between predicted wetland plant species richness and collection priority on the QTP, indicating that regions with higher potential wetland plant species richness should be prioritized for future sampling efforts (Figure 7). There was a significant positive correlation between priority areas for wetland plant collection on the QTP and inventory incompleteness, indicating that regions with inventory incompleteness of wetland plants on the QTP should be prioritized for collection in future efforts (Figure 8).

## 4 Discussion

### 4.1 Relationship Between Inventory Incompleteness of Wetland Plant and Accessibility Variables

It is generally assumed that the bias in biodiversity data collection at large spatial scales is due to the accessibility of transport facilities, especially in inaccessible areas such as some tropical regions (Herrera‐R et al. 2023). However, our research showed that the accessibility variable was not the most important factor influencing the incompleteness of wetland plants on the QTP. This is consistent with the findings of bias in freshwater fish collection in the Neotropics (Herrera‐R et al. 2023). In our study, we found an unexpected positive correlation between road density and inventory incompleteness. This contradicts previous studies, such as those in tropical East Africa and Myanmar, where inventory incompleteness was negatively correlated with road density and population density (Aung et al. 2023; Wang et al. 2020). Previous studies have often shown that higher road and population densities imply easier accessibility, resulting in greater intensity of plant specimen collection in these areas. In addition, specimen collection records tend to accumulate around specific regions rather than being randomly collected (Wang et al. 2020). The positive correlation between road density and inventory incompleteness is often observed in terrestrial plant communities, as samples are typically collected along and near roads, leading to higher sampling densities in these areas. In contrast, most of the wetlands involved in the collection of wetland plant specimens are located at some distance from roads, except for a small number of wetlands located along roads. Our study found a significant negative correlation between the shortest linear distance from wetlands to roads and the inventory incompleteness of wetland plants. This suggests that wetland plant specimens are more adequately collected when wetlands are closer to roads in terms of linear distance. However, the explained variance of inventory incompleteness by road distance (RDI) was limited, possibly due to its dependence on spatial scale. At higher resolution scales, the shortest linear distance from wetlands to roads results in a higher sampling density. Conversely, this relationship may show some decoupling at larger spatial scales (Yang et al. 2014).

Livestock density (LD) was a critical factor in predicting the inventory incompleteness of wetland plants. Wetlands provide abundant grazing resources and play an important role in the local livestock industry on the QTP. The Three Rivers Source Region, the Yarlung Zangbo River, the Nyangqu River, and the Lhasa River Region are important areas for animal husbandry and livestock breeding, with a relatively dense distribution of livestock in these regions (Zhan et al. 2023). Our results also indicated a relatively high degree of inventory incompleteness for wetland plants in these regions. Livestock prefer wetlands to uplands because of the abundance of grass and water (Zhan et al. 2023). Grazing can affect bank stability, increase sedimentation and siltation, and alter hydrological conditions. Changes in the habitat environment can lead to alteration, reduction, or even loss of wetland vegetation (Krall and Roni 2023). The species that thrive in these areas are often less sensitive to environmental change and may not accurately represent the full diversity of wetland plants in the region (de Almeida et al. 2021). For example, herbivores (such as cattle and sheep) consume most of the whole plant when grazing. This grazing behavior of herbivores can make it difficult to detect rare, low abundance wetland species during sample collection, thus affecting the collection of these taxonomic groups (Dai et al. 2022). On the other hand, livestock production on the plateau often takes the form of fencing. We have found that most pastures are isolated by wire fences, which prevents people from sampling wetlands. This can also lead to bias in the collection of wetland plant samples. Finally, areas with more livestock may have better natural conditions, such as abundant precipitation, which could lead to higher species richness. In regions with higher species diversity, it is more likely to have insufficient collection because more effort is needed for complete sampling.

### 4.2 Relationship Between Inventory Incompleteness of Wetland Plant and Attractiveness Variables

Our research showed that sample collection integrity was higher in protected areas than in regions disturbed by human activity, which is consistent with previous studies (de Araujo et al. 2022). Protected areas, which generally represent more conserved regions, may have higher species richness and a greater presence of rare taxa that attract the attention of botanists (de Almeida et al. 2021). In addition, sampling within the reserve will be guided and accompanied by staff, ensuring a higher level of safety (Wang et al. 2020). This study also found that regions with high wetland densities had more comprehensive sample collections, further confirming the importance of the botanist effect on sample collection. However, our study found that the inventory incompleteness of wetland plants was higher in regions with higher species richness, which contradicts previous research. In general, botanists are more likely to visit areas with high species richness (Wang et al. 2020). Biodiversity hotspots, such as the Hengduan Mountains, are characterized by a unique geological history and diverse habitat types, resulting in high species richness and endemism. Consequently, these regions are likely to be prioritized for extensive specimen collection (Yang et al. 2013, 2014). These regions have the potential to collect more species, leading to a higher chance of discovering rare and new species (Yang et al. 2014). This is also evidenced by the significant positive correlation between the collection priority of wetland plants identified in this study and the predicted species richness, suggesting that areas with higher potential species richness of wetland plants should be prioritized for future specimen collection on the QTP. However, in the relatively harsh and uniform climate of the north‐west region, the relatively low species richness and turnover make it very easy to achieve a relatively complete collection level with only a small number of specimens. As a result, the inventory incompleteness of wetland plants in these areas is relatively low (Yang et al. 2014). On the other hand, in the biodiversity hotspots of the southern and south‐eastern regions, although extensive sampling efforts have been made, the selection of many survey sites is often based on previously obtained classification and distribution data. This means that some areas within the hotspots are surveyed repeatedly, while many other sites remain under‐researched. Concentrated surveys by taxonomists do not aim to comprehensively reveal the biodiversity patterns of a region, but rather to collect taxa that are useful for comparison and phylogenetics, such as rare species. As a result, the places where rare groups are found often overlap with areas of high species richness (Sastre and Lobo 2009).

### 4.3 Relationship Between Inventory Incompleteness of Wetland Plant and Safety Variables

It is widely acknowledged that freshwater biodiversity surveys are frequently confronted with a unique set of logistical challenges (Herrera‐R et al. 2023). The climate of the QTP is variable, and the terrain is complex. The harsh environmental conditions pose a considerable challenge to field investigations. The findings of both linear regression and analysis of variance suggest that safety is the primary factor influencing the inventory incompleteness of wetland plants. As expected, inventory incompleteness is higher in the southern and south‐eastern regions of the plateau, where the risk of landslides is higher and the slopes are steeper. In order to conveniently collect specimens with identifiable reproductive structures (Panchen et al. 2019; Yang et al. 2021), wetland plant surveys on the QTP are usually conducted during the flowering and fruiting periods of plants. Due to the harsh environmental conditions on the plateau, the growing season for plants is quite short, typically lasting from May to October (Xie et al. 2020). At the same time, this period coincides with the peak occurrence of landslides in rivers and lakes on the plateau, along with abundant summer rainfall and snowmelt (Liu, Lu, et al. 2021), resulting in abundant river water and swift currents. The susceptibility to landslides is higher along the plateau's river basins, such as areas around rivers and roads, where landslides are common (Wang and Bai 2023). The inherent instability of riverbeds contributes to the complexity of the survey work. Consequently, in order to consider the safety of personnel engaged in the collection of wetland plant specimens, scientists frequently incorporate past occurrences of geological hazards, especially landslide risk indicators, into the formulation of more scientific and safer routes for field specimen collection.

In general, botanists prefer to conduct their work in rivers, lakes, and swamps with relatively gentle slopes, while wetlands with steep slopes and little safety are rarely visited. For example, the southeastern Yarlung Zangbo Grand Canyon in southeastern Tibet is recognized as one of the deepest and longest canyons in the world. The ecosystem in this area is diverse and complex, making it one of the world's biodiversity hotspots. However, the complex environmental conditions are major limiting factors for research on wetland plants in this area. In addition, inconvenient transportation (inaccessibility) is also a major factor leading to incomplete collection of wetland plant specimens in this area (Wu et al. 2022). In general, seasonality poses a challenge for large‐scale surveys of freshwater species (Herrera‐R et al. 2023). In seasonal wetlands, as water levels rise, small patches become submerged and, influenced by hydrology, merge to form larger marsh areas, which complicates access to sampling sites. At the same time, the flooding of seasonal water bodies in wetlands presents a safety risk for survey operations. Our research indicated a weak correlation between seasonal flooding and the inventory incompleteness of wetland plants. This could be due to the predictable nature of seasonal flooding on the QTP, allowing botanists to account for this when planning their survey routes, thus enhancing the safety of fieldwork (Herrera‐R et al. 2023).

Although the collection of wetland plant specimens on the QTP has been quite comprehensive, some gaps may still exist in the survey. Identifying priority collection areas based on weighted species richness can emphasize the significance of species with low collection numbers, as these species are often endemic or endangered (Wang et al. 2020). The priority collection areas for wetland plants identified in this study are situated in the southeastern region of the Plateau. This region is characterized by complex topography, substantial altitude variations, unstable geological formations, and a high incidence of geological hazards. Consequently, safety considerations are paramount when conducting specimen collection of wetland plants. With the advancement of unmanned aerial vehicle (UAV) technology, there is potential for addressing this issue. Du et al. (2021) utilized UAV hyperspectral images and three object‐ and pixel‐based machine‐learning classification algorithms to classify four dominant plant communities ( *Phragmites australis* , *Typha orientalis*, *Suaeda glauca*, and *Scirpus triqueter* ) in the Momoge Ramsar wetland. In this study, the discrepancies observed in the collection of wetland plant specimens may be attributed to limited data sources, including insufficient sharing of specimens and a lack of digitization by certain institutions (Wang et al. 2020). Furthermore, accurate identification of wetland plant specimens is a critical factor. For instance, recent years have seen few taxonomic revisions concerning the geographic distribution of various taxa, leading to provisional classifications and nomenclature for some groups. Additionally, several specimens remain misnamed, unidentified, or unclassified, persisting in this state for several decades (Goodwin et al. 2015).

However, we must acknowledge that an analytical bias is introduced by the data itself. Although we employed spatial autocorrelation analysis to mitigate the effects of data clustering, such clustering may still influence the identification of potentially rich and preferred collection areas in our analyses. This represents a limitation of the current study. In future research, we plan to implement a more systematic sampling scheme as the Second Integrated Scientific Expedition to the Tibetan Plateau progresses. Specifically, we will develop a standardized grid sampling scheme to ensure complete coverage of the entire study area through evenly distributed points. This improved sampling strategy aims to (1) reduce bias associated with uneven sample distribution, (2) improve the spatial representativeness of the data, and (3) provide more reliable baseline data for modeling. Furthermore, in future field surveys, we will concentrate on priority collection areas and utilize extensive field survey data to validate the predictions of the species distribution models developed in this study. This validation process will enable us to continually refine and enhance the models, thus increasing their accuracy. Ultimately, these improvements will establish a stronger foundation for the study and conservation of wetland plant diversity on the QTP.

## 5 Conclusions

In this study, we assessed the inventory incompleteness of wetland plants across various regions of the QTP and identified the factors contributing to discrepancies in wetland plant sample collection. Our findings indicate that the inventory completeness of wetland plants on the QTP is relatively high; however, a significant degree of incompleteness was observed in the southern and southeastern regions compared to other areas of the plateau. Safety emerged as the most critical factor influencing inventory incompleteness, while accessibility and attractiveness also played significant roles. Among these factors, landslide risk, slope, livestock density, and species richness were identified as the most significant predictors of inventory incompleteness. This study provides valuable insights into the limitations of wetland ecosystems and serves as an important reference for future wetland plant sampling and conservation efforts on the QTP.

## Author Contributions

**Yigang Li:** data curation (lead), formal analysis (lead), methodology (lead), writing – original draft (lead), writing – review and editing (lead). **Fan Liu:** formal analysis (equal), investigation (equal), methodology (equal), writing – review and editing (equal). **Meiying Gong:** data curation (equal), formal analysis (equal), methodology (equal), writing – review and editing (equal). **Xing Liu:** data curation (equal), funding acquisition (lead), investigation (equal), methodology (equal), writing – review and editing (equal). **Changchun Li:** conceptualization (equal), methodology (equal), writing – review and editing (equal).

## Conflicts of Interest

The authors declare no conflicts of interest.

## Supporting information


Appendix S1

Appendix S2

Appendix S3

Figure S1

## Data Availability

This study used published data, which were cited in the article. Data supporting the analyses reported in this study are available in Supporting Information.
