# Supplementary material for: The Combination of Safety, Attractiveness, and Accessibility Lead to Bias in Inventory of Wetland Plants on the Qinghai‐Tibet Plateau
Source: Ecol Evol. 2025 Jun 4;15(6):e71521. doi: 10.1002/ece3.71521 (PMC12137625; doi:10.1002/ece3.71521)
Supplement: Supplementary file 3 — Appendix S3 [file ECE3-15-e71521-s002.docx]

Appendix S3. Pearson correlation analyses among explanatory variables for inventory incompleteness of wetland plants on the Qinghai-Tibet Plateau, including four accessibility variables, three attractiveness variables, and three safety variables. ^*^ *p* < 0.05; ^**^ *p* < 0.01; ^***^ *p* < 0.001

|  | LR | Slope | IE | WA | NA | SR | LD | RDE | HD | RDI |
| --- | --- | --- | --- | --- | --- | --- | --- | --- | --- | --- |
| LR | 1.00 |  |  |  |  |  |  |  |  |  |
| Slope | 0.68^***^ | 1.00 |  |  |  |  |  |  |  |  |
| IE | 0.25^***^ | 0.25^***^ | 1.00 |  |  |  |  |  |  |  |
| WA | -0.26^***^ | -0.42^***^ | -0.29^***^ | 1.00 |  |  |  |  |  |  |
| NA | -0.25^***^ | -0.32^***^ | -0.07 | 0.07 | 1.00 |  |  |  |  |  |
| SR | 0.81^***^ | 0.66^***^ | 0.27^***^ | -0.18^**^ | -0.18^**^ | 1.00 |  |  |  |  |
| LD | 0.67^***^ | 0.58^***^ | 0.16^*^ | 0.08 | -0.33^***^ | 0.63^***^ | 1.00 |  |  |  |
| RDE | 0.71^***^ | 0.50^***^ | 0.10 | -0.10 | -0.33^***^ | 0.63^***^ | 0.71^***^ | 1.00 |  |  |
| HD | 0.74^***^ | 0.58^***^ | 0.32^***^ | -0.17^**^ | -0.31^***^ | 0.66^***^ | 0.78^***^ | 0.76^***^ | 1.00 |  |
| RDI | -0.35^***^ | -0.41^***^ | -0.09 | 0.05 | 0.32^***^ | -0.39^***^ | -0.57^***^ | -0.59^***^ | -0.50^***^ | 1.00 |

Abbreviations：LR: landslide risk, Slope: slope, IE: inundation extent, WA: wetland area, NR: nature reserve area, SR: predicted species richness, LD: livestock density, RDE: road density, HD: human population density, RDI: road distance.
