# Supplementary material for: The Combination of Safety, Attractiveness, and Accessibility Lead to Bias in Inventory of Wetland Plants on the Qinghai‐Tibet Plateau
Source: Ecol Evol. 2025 Jun 4;15(6):e71521. doi: 10.1002/ece3.71521 (PMC12137625; doi:10.1002/ece3.71521)
Supplement: Supplementary file 4 — Figure S1 [file ECE3-15-e71521-s003.docx]

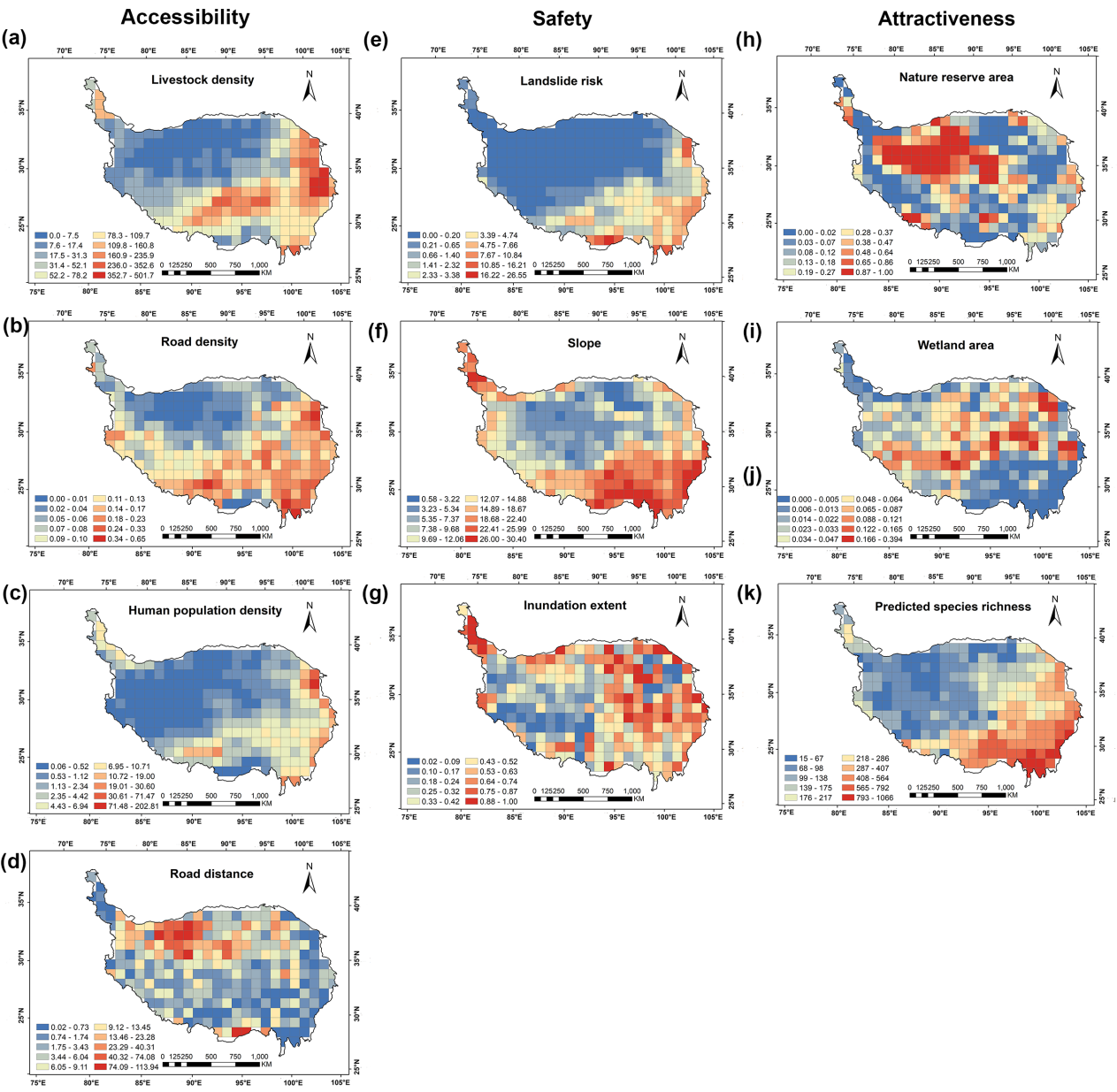


**FIGURE S1** Geographical patterns of explanatory variables for inventory incompleteness of wetland plants on the Qinghai-Tibet Plateau based on a 100 km × 100 km grid., including four accessibility variables, three attractiveness variables, and three safety variables.
